# Conditional gene deletion reveals functional redundancy of GABA_B _receptors in peripheral nociceptors *in vivo*

**DOI:** 10.1186/1744-8069-5-68

**Published:** 2009-11-19

**Authors:** Vijayan Gangadharan, Nitin Agarwal, Stefan Brugger, Imgard Tegeder, Bernhard Bettler, Rohini Kuner, Martina Kurejova

**Affiliations:** 1 Pharmacology Institute, University of Heidelberg, Im Neuenheimer Feld 366, 69120 Heidelberg, Germany; 2 pharmazentrum frankfurt/ZAFES, Goethe-University Clinic Frankfurt am Main, Theodor-Stern-Kai 7, 60590 Frankfurt am Main, Germany; 3 Department of Biomedicine, Pharmazentrum, University of Basel, Klingelbergstrasse 50/70, CH-4056 Basel, Switzerland; 4 Anesthesiology and Intensive Medicine, University Hospital of Basel, Switzerland

## Abstract

**Background:**

γ-aminobutyric acid (GABA) is an important inhibitory neurotransmitter which mainly mediates its effects on neurons via ionotropic (GABA_A_) and metabotropic (GABA_B_) receptors. GABA_B _receptors are widely expressed in the central and the peripheral nervous system. Although there is evidence for a key function of GABA_B _receptors in the modulation of pain, the relative contribution of peripherally- versus centrally-expressed GABA_B _receptors is unclear.

**Results:**

In order to elucidate the functional relevance of GABA_B _receptors expressed in peripheral nociceptive neurons in pain modulation we generated and analyzed conditional mouse mutants lacking functional GABA_B(1) _subunit specifically in nociceptors, preserving expression in the spinal cord and brain (SNS-GABA_B(1)_^-/- ^mice). Lack of the GABA_B(1) _subunit precludes the assembly of functional GABA_B _receptor. We analyzed SNS-GABA_B(1)_^-/- ^mice and their control littermates in several models of acute and neuropathic pain. Electrophysiological studies on peripheral afferents revealed higher firing frequencies in SNS-GABA_B(1)_^-/- ^mice compared to corresponding control littermates. However no differences were seen in basal nociceptive sensitivity between these groups. The development of neuropathic and chronic inflammatory pain was similar across the two genotypes. The duration of nocifensive responses evoked by intraplantar formalin injection was prolonged in the SNS-GABA_B(1)_^-/- ^animals as compared to their control littermates. Pharmacological experiments revealed that systemic baclofen-induced inhibition of formalin-induced nociceptive behaviors was not dependent upon GABA_B(1) _expression in nociceptors.

**Conclusion:**

This study addressed contribution of GABA_B _receptors expressed on primary afferent nociceptive fibers to the modulation of pain. We observed that neither the development of acute and chronic pain nor the analgesic effects of a systematically-delivered GABA_B _agonist was significantly changed upon a specific deletion of GABA_B _receptors from peripheral nociceptive neurons *in vivo*. This lets us conclude that GABA_B _receptors in the peripheral nervous system play a less important role than those in the central nervous system in the regulation of pain.

## Background

Metabotropic GABA receptors, namely GABA_B _receptors, mediate the slow and prolonged physiological effects of the inhibitory neurotransmitter, GABA. They play an important role in the modulation of synaptic transmission. Contribution of pre- as well as post-synaptic GABA_B _receptors in the modulation of long-term plasticity phenomena in brain regions, such as the hippocampus and amygdala, has been described [1-5]. GABA_B _receptors are also highly concentrated in the superficial dorsal horn, predominantly on afferent terminals of sensory neurons located in the dorsal root ganglia (DRG) [6-9]. Amongst these, small-diameter nociceptive neurons show a high density of GABA_B _receptor expression [7,10]. However, GABA_B _receptors are also expressed postsynaptically on second order neurons and as well as at motor neuron synapses [11,12]. The expression of GABA_B _receptor subunits is enhanced in lumbal spinal cord and dorsal root ganglion following chronic nociceptive activation in models of axotomy and chemogenic pain [13].

Multiple lines of evidence support an antinociceptive role for GABA_B _receptors in animal models of acute and chronic pain. baclofen, a GABA_B _receptor agonist, exhibits antinociceptive effects in model of peripheral nerve injury and chronic inflammation [14]. baclofen also attenuates pain-related behaviors evoked by the formalin injection in rats and also reduces allodynia-like behavioral symptoms in disease models of chronic pain inducing monoarthritis [15], ischemic injury to the spinal cord [16], carrageenan-produced inflammation [17] or trigeminal neuralgia [18,19]. In the view of extensive literature in animal models of acute and chronic pain, it is rather surprising that the clinical administration of GABA_B _receptor agonists as analgesics has been restricted to trigeminal neuralgia and post-herpetic neuralgia [20,21]. Indeed, GABA_B _receptor agonists display muscle relaxant properties and are rather widely used in the control of spasticity [22,23] and have been implicated in dystonia [24]. Because evoked pain behaviors in animal studies mostly rely upon a motor behavioral response, the motor deficits caused by GABA_B _receptor modulation occlude an unequivocal interpretation of behavioral responses. Another important caveat is that currently available GABA_B _ligands suffer from lack of selectivity with respect to the locus of action within the different components of the spinal sensory-motor circuit. Thus, a complete delineation of the sensory antinociceptive actions from the motor inhibitory actions of GABA_B _receptors is not possible using the conventional approach of ligand delivery in animals.

We reasoned that the application of genetic tools to manipulate GABA_B _receptor expression in a site-specific manner may enable delineating their specific role in the modulation of nociception and chronic pain. We generated mice lacking the GABA_B _receptors specifically in nociceptive neurons of the dorsal root ganglia. Detailed behavioral and electrophysiological analyses in the paradigms of basal and pathological nociception revealed that although GABA_B _receptors localized in first order nociceptive neurons have the capacity to modulate sensitization phenomena, their contribution towards the modulation of pain at the level of the whole living organism is not pronounced. Furthermore, pharmacological experiments showed that baclofen-induced antinociception is mechanistically-independent of GABA receptors in the first order nociceptive neurons.

## Materials and methods

### Genetically-modified mice

Homozygous mice carrying the *GABAB1 *flox allele (GABA_B(1) _flanked by loxP sites, GABA_B(1)_^fl511/fl511^) have been described previously in details [25] GABA_B(1)_^fl511/fl511 ^mice were crossed with SNS-Cre mice [26] to obtain GABAB1-LoxPSNS-Cre+ mice (referred to as SNS-GABA_B(1)_^-/- ^mice in this manuscript) and GABA_B(1)_^fl/fl ^mice (control littermates). Genotyping was done on mouse genomic tail DNA using primers: for sense strand 5'-ATCTCTTCCTTGGCTGGGTCTTTGCTTCGCTCG-3' and for anti-sense 5'-GGGTTATTGAATATGATCGGAATTCCTCGACT-3' to detect *GABAB1 *flox allele, and for sense strand 5'-GAAAGCAGCCATGTCCAATTTACTGACCGTAC-3' and for anti-sense strand 5'-GCGCGCCTGAAGATATAGAAGA-3' to detect SNS-Cre transgene expression. Both, SNS-Cre and GABA_B(1)_^fl511/fl511 ^mice were backcrossed individually into their background for more than 8 generations before being crossed with each other. Littermates were used in all experiments to control for background effects.

### Western blotting

Western blots were performed with lysates of mouse DRG, spinal cord and brain with antibodies recognizing murine GABA_B(1) _(AB1531, Chemicon) and alpha-tubulin (Sigma Aldrich) according to standard protocols [27].

### In Situ Hybridisation

For generation of riboprobes, 1.7 kb-long GABA_B(1)_-specific probes were generated and *in situ *hybridisation using nonradioactive Dig-UTP-labelled antisense and sense probes was performed on cryostat sections of DRG (16 μm) as described in details previously [28].

### Nociceptive tests and mouse models of pain

All animal use procedures were in accordance with ethical guidelines imposed by the local governing body (Regierungspräsidium Karlsruhe, Germany). All behavioral measurements were done in awake, unrestrained, age-matched, female or male mice which were more than 3 months of age. Complete Freund's adjuvant (CFA, Sigma Aldrich) was injected unilaterally in the intraplantar surface of the mouse hindpaw (20 μl) as described in details previously [29]. Analysis of latency of paw withdrawal in response to noxious heat was done as described in details [29] (Ugo Basile Inc.). Mechanical sensitivity was tested in the same group of animals using von Frey hairs.

The 'spared nerve injury' (SNI) model for neuropathic pain was performed as described in details previously [30]. Two of the three terminal branches of the sciatic nerve (tibial and common peroneal nerves) were ligated and cut leaving the remaining third branch (sural nerve) intact. Mechanical allodynia following SNI was measured as paw withdrawal latency to dynamic von Frey stimulation (Ugo Basile, maximum force 5 g, ramp 10 sec) [31]. Response to thermal stimulus (52°C) in the SNI model was done via a hot plate latency test. The latency to paw licking, paw withdrawal or jumping after placing the animal to the hot plate was measured. Cold allodynia following SNI was assessed by counting the number of responses (flinching, licking, jumping, and shaking) on a 5°C cold plate during an observation period of 90 seconds. Additionally, cold allodynia was determined by measuring the duration of paw licking, lifting and flinching in response to plantar application of a drop of acetone. The nociceptive behavior was observed for 90 sec starting immediately after acetone application.

Formalin (1%, 20 μl) was injected into the plantar surface of the hindpaw and the duration of nocifensive behaviors (lifting, licking, or flinching) was measured in 5 min bins for 60 min after formalin injection as described previously [32]. baclofen (2 mg/kg of body weight; Lioresal intrathecal, Novartis) or sterile PBS for control animals were injected intraperitonially 30 min before 1% formalin administration.

### Afferent recordings in skin-nerve preparation

A total of 19 mice (10 GABA_B(1)_^fl/fl ^and 9 SNS-GABA_B(1)_^-/-^) were used in the electrophysiological investigations. An *in vitro *skin nerve preparation was used to study the properties of mechanosensitive C- and Aδ-afferent fibers which innervate the skin in the inflamed area. Experiments were performed on the dissected skin of control animals and animals sacrificed 4 hours following CFA inoculation into the hindpaw (30 μl). Animals were killed by CO_2 _inhalation, the saphenous nerve was dissected with the skin of the dorsal hind-paw attached and mounted in an organ bath "inside-up" to expose the dermis. The preparation was perfused with an oxygen-saturated modified synthetic interstitial fluid solution containing (in mM) 123 NaCl, 3.5 KCl, 0.7 MgSO_4_, 1.5 NaH_2_PO_3_, 1.7 NaH_2_PO_4_, 2.0 CaCl_2_, 9.5 sodium gluconate, 5.5 glucose, 7.5 sucrose, 10 Hepes at temperature of 32 ± 1°C and pH 7.4 ± 0.05. Fine filaments were teased from the desheathed nerve placed in separate chamber and placed on a recording electrode.

Nerve fibers were classified according to their conduction velocities, von Frey thresholds, and firing properties. Electrical stimulation of the nerve fiber was employed to calculate conduction velocities of individual nerve fibers. Fibers which conducted < 1 m/s and fibers conducting between 1-10 m/s were considered to be unmyelinated C-fibers and myelinated Aδ-fibers, respectively. Some of the fibers of the velocities around 1 m/s where not included into the analyses if further detailed classification according to the firing properties and threshold was not possible.

Once the receptive field was identified using the glass rod a computer-controlled linear stepping motor (Nanomotor Kleindiek Nanotechnik) [33] was used to apply standardized mechanical stimuli. Each fiber was tested with series of displacement mechanical stimuli ranging from 6 to 384 μm for both control and CFA injected animals. Electrophysiological data were collected with Powerlab 4.0 system and analyzed off-line with the spike histogram extension of the software.

### Data analysis & statistics

All data are presented as mean ± standard error of the mean (S.E.M.). Analysis of variance (ANOVA) for random measures was performed followed by post-hoc Fisher's test to determine statistically significant differences. p < 0.05 was considered significant.

## Results

### Conditional and specific deletion of GABA_B(1) _in nociceptors

We utilized the Cre-loxP system to generate mice conditionally lacking GABA_B _receptors specifically in primary nociceptors. We have previously described the generation of BAC transgenic mice expressing the Cre-recombinase under the influence of promoter elements of the mouse *Scn10a *gene encoding the Na_V_1.8 sodium channel. This line enables gene deletion specifically in c- and Aδ-nociceptors in the dorsal root ganglia while preserving gene expression in the central nervous system and other tissues [26,34]. Mice carrying floxed *GABAB1 *allele [25] were crossed with SNS-Cre+ mice to generate homozygous mice in which the Cre-mediated excision of *GABA*_B(1) _exon will assure the absence of *GABAB1 *gene product in pre-synaptic order nociceptive neurons and their peripheral and central terminals. These include all of the known C-terminal variants of GABA_B(1)_, such as GABA_B(1a)_, which contributes largely to the GABA_B(1) _expression in nociceptors, GABA_B(1b)_, and the additional, newly described GABA_B(1e)_, that can mediate dominant-negative effects on GABA_B _receptor heteromerization and is indeed expressed in pain pathways [35]. Based on our understanding of GABA_B _receptor heteromerization, lack of the GABA_B(1) _subunit precludes the assembly of functional GABA_B _receptor [28,36-38]. Previous studies have shown that the deletion of GABA_B(1) _subunit is sufficient to cause the loss of pre- and post-synaptic GABA_B _responses [25,39].

**Figure 1:**
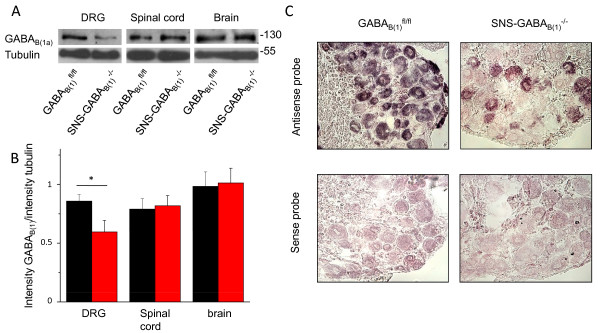
**Conditional deletion of GABA_B(1) _receptor in sensory neuron specific GABAB1 knockout mice (SNS-GABA_B(1)_^-/-^)**. (A) Western blot analysis of dorsal root ganglion (DRG), spinal cord and brain of GABA_B(1)_^fl/fl ^and SNS-GABA_B(1)_^-/- ^using anti-GABA_B(1) _antibody. Equal loading of samples was controlled via analysis of tubulin expression. (B) Quantitative analysis of western blot experiments. Shown are ratios of signal intensity for GABA_B(1) _normalized to signal intensity of tubulin expression (n = 3; p < 0.05; ANOVA). (C) mRNA *in situ *hybridization for expression of *GABAB1 *in DRG sections from GABA_B(1)_^fl/fl ^and SNS-GABA_B(1)_^-/-^. Antisense mRNA riboprobes showed loss of signal in small-diameter DRG neurons in SNS-GABA_B(1)_^-/- ^in comparison to their GABA_B(1)_^fl/fl ^littermates, whereas expression was retained in large-diameter cells.

Western blot analysis showed that in SNS-GABA_B(1)_^-/- ^mice expression of GABA_B(1) _protein in the dorsal root ganglia (DRG) is largely reduced, whereas expression in the brain and spinal cord remains unchanged (Fig. 1A and 1B). *In situ *mRNA hybridisation using GABA_B(1)_-specific riboprobes revealed that only a small proportion of neurons expressing mRNA for GABA_B(1) _belongs to the group of large diameter neurons. A majority of GABA_B(1) _expressing DRG neurons are small-diameter neurons. SNS-GABA_B(1)_^-/- ^mice showed a selective deletion of GABA_B(1) _in small diameter neurons of the DRG whereas expression in large-diameter neurons was preserved (Fig. 1C).

### Electrophysiological analyses of peripheral nerve activity in SNS-GABA_B(1)_^-/- ^mice

To clarify the specific contribution of GABA_B _receptors on peripheral terminals, we performed electrophysiological recordings on peripheral polymodal C-fiber nociceptors and Aδ nociceptors, which were identified on the basis of stimulation and conduction properties in a hindpaw skin-nerve preparation [33]. The skin was dissected from both genotypes from either naive mice or 4 hours after injection of 30 μl CFA into the hindpaw.

**Figure 2:**
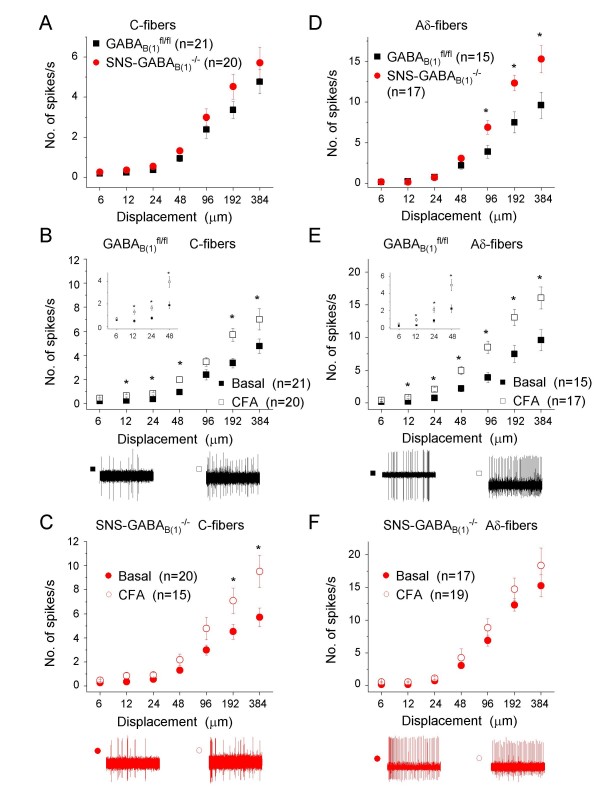
**Electrophysiological analysis of excitability of peripheral nociceptive fibers in GABA_B(1)_^fl/fl ^and SNS-GABA_B(1)_^-/- ^mice in the skin nerve preparation**. Shown are electrophysiological recordings of spike rates evoked by application of pressure via a nanomotor (expressed in terms of displacement) from c-mechanoceptors (C-fibers, panel A, B and C) and Aδ-type of mechanoceptors (panel D, E and F) in the skin-nerve preparation derived from the paws of GABA_B(1)_^fl/fl ^and SNS-GABA_B(1)_^-/- ^mice. Panel A and D represent analyses in naive GABA_B(1)_^fl/fl ^and SNS-GABA_B(1)_^-/-^. Panels B and E represent analyses in GABA_B(1)_^fl/fl ^mice at 4 h following CFA-induced hindpaw inflammation. Insets show the magnification of the firing properties for displacements from 6 to 48 μm. Panel C and E represent analyses in SNS-GABA_B(1)_^-/- ^mice at 4 h following CFA-induced hindpaw inflammation. Representative traces of firing properties of C-fibers and Aδ-mechanoceptors for nanomotor displacement of 48 μm are shown below the quantitative summaries. * indicates significant statistical difference (p < 0.05; ANOVA followed by post-hoc Fisher's test). n represents the number of fiber type for each tested animal group.

Recordings were made from C- and Aδ-fibers because these neurons are targeted by the SNS-Cre-mediated deletion of GABA_B(1)_. Stimulus-response functions of C-fibers from naive SNS-GABA_B(1)_^-/- ^mice and GABA_B(1)_^fl/fl ^mice demonstrated no significant changes between the responsiveness of this population to mechanical stimulation (Fig. 2A). At 4 h following CFA-induced hind paw inflammation, the excitability of mechanoreceptive C-fibers increased significantly in GABA_B(1)_^fl/fl ^mice (Fig. 2B; p < 0.005, ANOVA followed by posthoc Fisher's test; typical examples of traces are shown below the quantitative summary in Fig. 2B). Mechanoreceptive C-fibers of SNS-GABA_B(1)_^-/- ^mice also demonstrated increased excitability following CFA-induced inflammation; however, statistical significance was only reached at higher forces of mechanical stimulation. Surprisingly, analysis of the basal activity of mechanoreceptive AM-fibers (Aδ-fibers) revealed that the basal excitability is significantly increased in mice lacking GABA_B(1) _in peripheral nociceptors (Fig. 2D) whereas GABA_B(1)_^fl/fl ^mice demonstrated strong hyperexcitability to the entire range of intensities of mechanical stimuli employed following CFA-induced inflammation (Fig. 2E). Aδ-fibers in SNS-GABA_B(1)_^-/- ^mice failed to demonstrate any further increases in excitability following CFA (Fig. 2F). The magnitude of responses shown by SNS-GABA_B(1)_^-/- ^mice to various mechanical forces in the naive state was equivalent to those shown by the GABA_B(1)_^fl/fl ^mice in the CFA-induced sensitized state and no further sensitization was then seen in SNS-GABA_B(1)_^-/- ^mice following CFA-induced inflammation.

### Development of acute nociceptive hypersensitivity and its pharmacological modulation by baclofen

**Figure 3:**
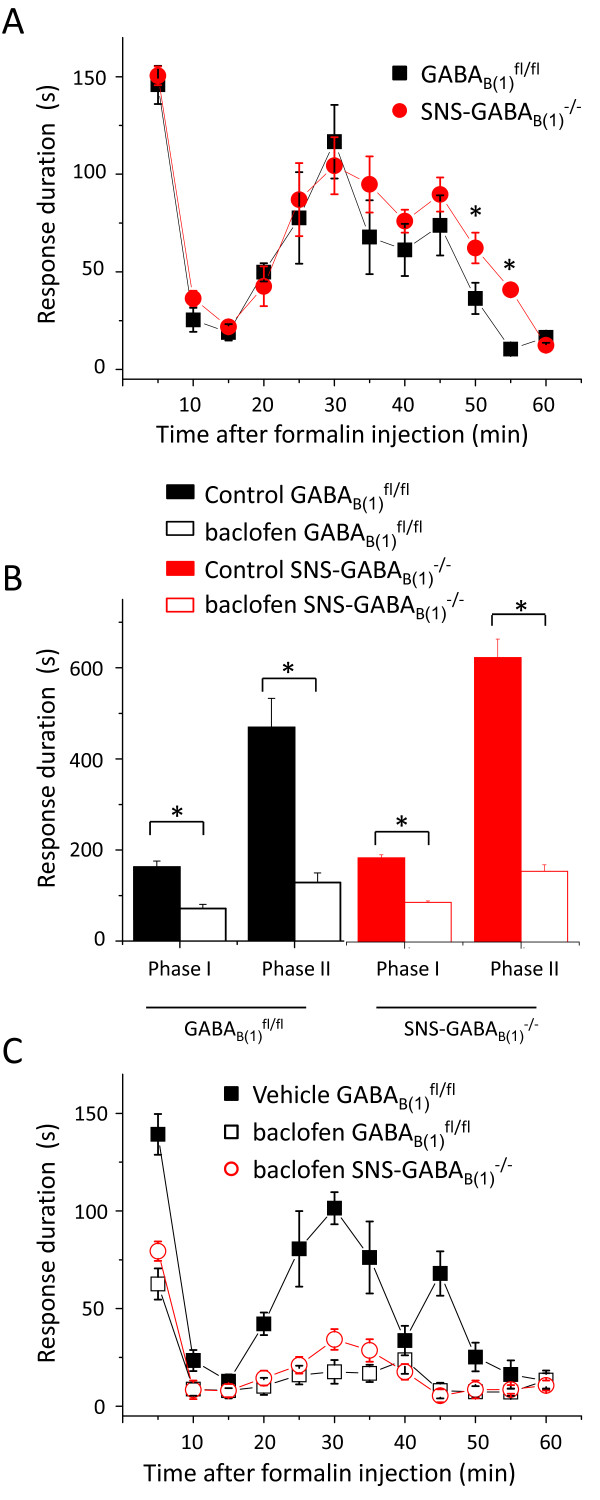
**Formalin-induced nociception and its modulation by GABA_B(1) _agonist, baclofen, in GABA_B(1)_^fl/fl ^and SNS-GABA_B(1)_^-/- ^mice**. (A) Time course of nocifensive behavior after the injection of 1% formalin into the hindpaw in SNS-GABA_B(1)_^-/- ^mice and their control littermates. (B) Cumulative analysis of phase I (0-10 min) and phase II (10-60 min) of the formalin response shows no significant differences in both genotypes, SNS-GABA_B(1)_^-/- ^and GABA_B(1)_^fl/fl ^mice. Intraperitoneal injection of baclofen (2 mg/kg body weight) applied 30 minutes before intraplantar injection of formalin caused a reduction in both phases in GABA_B(1)_^fl/fl ^as well as SNS-GABA_B(1)_^-/- ^mice. (C) baclofen-induced modulation of the time course of the formalin responses foregoing by intraperitoneal injection of baclofen in GABA_B(1)_^fl/fl ^and SNS-GABA_B(1)_^-/-^. PBS was injected in the control group. * indicates significant statistical difference (p < 0.05; ANOVA followed by post-hoc Fisher's test).

To determine how GABA_B _receptor affects behavioral correlates of rapid sensitization in pain pathways, we performed the plantar formalin test on SNS-GABA_B(1)_^-/- ^mutant mice and their wild-type controls [32]. Intraplantar injection of formalin in rodents evokes nocifensive behaviors such as licking, shaking, and lifting of the injected paw in a biphasic manner. Phase I of the formalin response (0-10 min after injection) is caused by persistent activation and acute sensitization of nociceptors, whereas the phase II response (10-60 min after injection) result from continual activation of nociceptors and a sensitization of central synapses via mechanisms which are triggered by repetitive stimulation during the first phase [32]. GABA_B(1) _mutant mice failed to reveal changes in the total cumulative duration of phase I and phase II responses to formalin injection (1%, 20 μl) (Fig. 3B). However, the latter part of the phase II formalin response (so called phase IIb, [40]) was significantly prolonged in SNS-GABA_B(1)_^-/- ^(n = 5) when compared to GABA_B(1)_^fl/fl ^animals (n = 5) (Fig. 3A).

Previous studies on formalin-induced nocifensive behavior showed that intraperitoneal or intrathecal injection of GABA-B receptor agonist, baclofen induces antinociception in both phases of the formalin test [41,42]. Consistent with previous studies, baclofen (2 mg/kg of body weight), administered intraperitoneally 30 min prior to the injection of formalin showed antinociceptive effect on phase I and II (Fig. 3B, C; p < 0.05) behavior. However, baclofen was equally efficient in depressing nociceptive behavior on SNS-GABA_B(1)_^-/- ^(n = 5) and GABA_B(1)_^fl/fl ^(n = 5) mice (Fig. 3B, C; p < 0.05) suggesting that the effect of baclofen is independent on expression of GABA_B _receptors in peripheral nervous system.

### Development of chronic inflammatory pain in SNS-GAB_B(1)_^-/- ^mice

**Figure 4:**
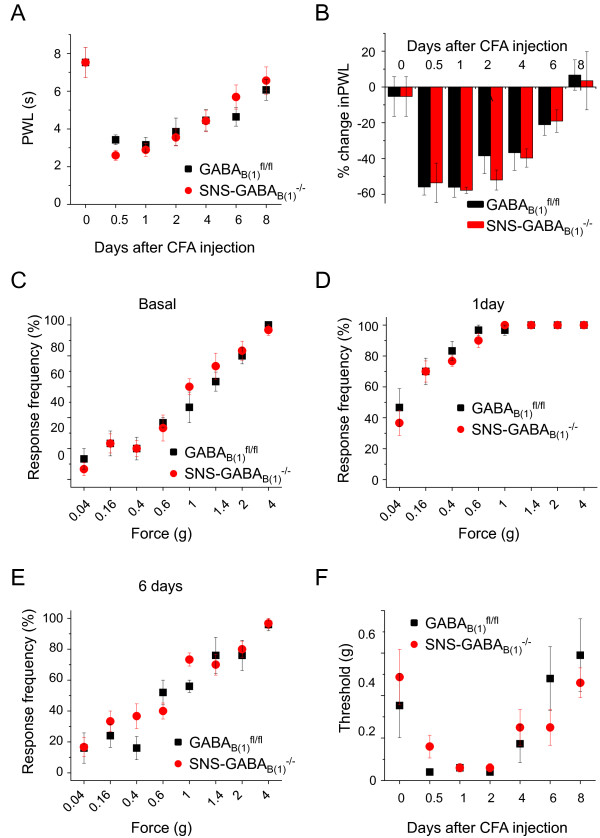
**Behavior analysis of GABA_B(1)_^fl/fl ^and SNS-GABA_B(1)_^-/- ^mice in the CFA model of inflammatory pain**. (A) Changes in paw withdrawal latency (in s) in response to noxious heat 12 hours and 1, 2, 4, 6 and 8 days following unilateral intraplantar injection of CFA. (B) Thermal hyperalgesia (changes in latency of paw withdrawal in response to noxious heat applied to the hindpaw plantar surface either before (basal) or at 12 h, and 1, 2, 4, 6 and 8 days) following unilateral intraplantar injection of CFA. Y axes represent the percent difference in paw withdrawal latency between the injected and uninjected paws calculated as (injected paw - uninjected paw) × 100/uninjected paw (negative values therefore indicate hyperalgesia). Comparison of response frequency to von Frey hairs in GABA_B(1)_^fl/fl ^(n = 6) and SNS-GABA_B(1)_^-/- ^(n = 6) mice prior to (C), 1 days (D) and 6 days (E) following intraplantar injection of CFA. (F) Summary of threshold (defined as a force eliciting a response frequency of at least 40%) prior to and at 12 hours and 1, 2, 4, 6 and 8 days following intraplantar injection of CFA.

Development of somatic inflammatory pain and hyperalgesia was assessed in GABA_B(1)_^fl/fl ^mice and SNS-GABA_B(1)_^-/-^mice at 12 h, 1, 2, 4, 6 and 8 days following unilateral hindpaw inflammation induced by intraplantar injection of complete Freud's adjuvant (CFA; 20 μl). In CFA-injected animals paw withdrawal latency (PWL) to noxious radiant heat decreased significantly for up to 4 days (Fig. 4A). CFA-induced thermal hyperalgesia was comparable in SNS-GABA_B(1)_^-/- ^(n = 6) and GABA_B(1)_^fl/fl ^(n = 6) mice (Fig. 4B). Magnitudes of mechanical hyperalgesia that developed after CFA injection was tested using von Frey filaments on SNS-GABA_B(1)_^-/- ^mice, and their respective control littermates (Fig. 4C-F). Upon CFA injection, no significant variance in the magnitudes of both allodynia (defined as responses to 0.16-0.4 g force) as well as mechanical hyperalgesia (defined as responses to 0.6 - 4 g force) was observed in SNS-GABA_B(1)_^-/- ^(n = 6) mice compared to GABA_B(1)_^fl/fl ^mice (n = 6) (Fig. 4C-E). No significant difference in the relative drop in response thresholds that is defined as minimum force required to elicit 40% response frequency over the basal state was observed between genotypes (Fig. 4F). These results imply that a loss of GABA_B _receptors in nociceptive DRG neurons does not produce a major effect on nociceptive behavior and is not involved in the modulation of chronic inflammatory pain.

### Development of neuropathic pain in SNS-GAB_AB(1)_^-/- ^mice

**Figure 5:**
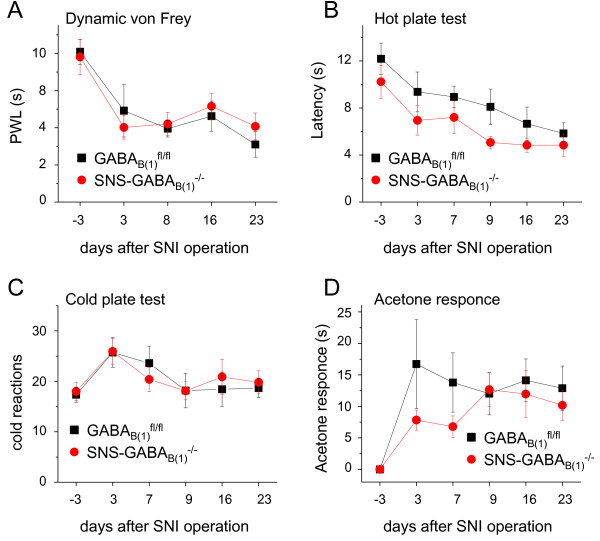
**Analysis of GABA_B(1)_^fl/fl ^and SNS-GABA_B(1)_^-/- ^in the Spared nerve injury model for neuropathic pain**. (A) Latency of paw withdrawal to dynamic von Frey stimulation in GABA_B(1)_^fl/fl ^and SNS-GABA_B(1)_^-/- ^mice before and 3,7,9,16 and 23 days following spared nerve injury (SNI). (B) Latency of thermal responses via hot plate latency test (52°C) (C) Number of reactions to a 5°C cold stimulus (flinching, licking, jumping, and shaking) during an observation period of 90 seconds on a cold plate. (D) Response to plantar application of acetone in GABA_B(1)_^fl/fl ^and SNS-GABA_B(1)_^-/-^. All data points represent mean ± SEM. Statistical significance was not reached between mice of the two genotypes (ANOVA).

To assess whether peripheral GABA_B _receptors play a role in nociceptive hypersensitivity which develops following a peripheral nerve lesion, we employed the spared nerve injury model (SNI) of neuropathic pain [30]. At any of the time points studied following injury (3, 7, 9, 16 and 23 days), no differences were found with respect to hyperalgesia and allodynia between SNS-GABA_B(1)_^-/- ^mice and GABA_B(1)_^fl/fl ^mice. Following SNI, profound mechanical allodynia was recorded by measuring paw withdrawal latency using a dynamic aesthesiometer. Mechanical allodynia developed to a similar extent in SNS-GABA_B(1)_^-/- ^and GABA_B(1)_^fl/fl ^mice (Fig. 5A). Thermal hyperalgesia to heat developed to a comparable extent in GABA_B(1)_^-/- ^mice and GABA_B(1)_^fl/fl ^mice (Fig. 5B). Similarly, cold allodynia (responses to 5°C) developed equally in SNS-GABA_B(1)_^-/- ^mice and GABA_B(1)_^fl/fl ^mice following SNI (Fig. 5C, D). From these data, we infer that GABA_B _receptors expressed by Aδ- and C-nociceptive peripheral neurons are not involved in regulation of neuropathic pain.

## Discussion

A large body of morphological, electrophysiological, behavioral, and pharmacological studies have implicated GABA_B _receptors in the control of pain. However, owing to the broad expression of GABA_B _receptors throughout the nervous system, spatially and temporally restricted manipulation of GABA_B _receptor expression is needed to elucidate their role at different anatomical sites along the pain pathway. In this study, we generated mice lacking GABA_B(1) _receptor specifically in the peripheral arm of the nociceptive pathway. These mice are well-suited for elucidating the relevance of GABA_B _receptor-mediated presynaptic inhibition of neurotransmitter release from nociceptor terminals as well as a putative role for GABA_B _receptors in peripheral nociceptive terminals in physiological and pathophysiological states. We analyzed mice with respect to the excitability of nociceptors and its manifestations in several models of pain, including unilateral hindpaw inflammation, chemogenic activation of nociceptors and peripheral neuropathy. Surprisingly, our detailed analyses revealed very few phenotypic differences between mice lacking GABA_B _receptors in nociceptors and control mice. Briefly, our main findings were: 1. Chemogenic pain evoked by formalin and early nociceptive hypersensitivity were slightly prolonged in SNS-GABA_B(1)_^-/- ^mice. 2. The magnitude and duration of chronic inflammatory pain and neuropathic pain was comparable between SNS-GABA_B(1)_^-/- ^mice and control littermates. 3. Electrophysiological analyses of nociceptor activity revealed a higher basal excitability in Aδ-mechanoceptors in SNS-GABA_B(1)_^-/- ^mice; however, this did not translate into clear functional changes with respect to nociceptive behavior.

Our findings are surprising in the view of previous studies reporting GABA_B _receptor expression in primary afferent terminals [6-9] as well as functional studies which show that GABA_B _receptor activation on primary afferent terminals in the spinal cord reduces neurotransmitter release [43-45]. Although it is clear that GABA_B _receptors are densely expressed in peripheral nociceptive neurons, the literature on the regulation of GABA_B _receptor expression in pathological pain states is somewhat mixed. For example, some studies reported an increase in GABA_B _receptor expression in the spinal dorsal horn and peripheral nociceptors in inflammatory pain states [13]. In contrast, Engle et al. [46] found that spinal nerve ligation does not alter the expression or function of GABA_B(1) _and GABA_B(2) _in the spinal cord and dorsal root ganglia of rats and also does not lead to changes in GABA_B _receptor binding affinity in inflammatory and neuropathic states. Furthermore, findings in a model of diabetic neuropathy suggest reduced function of presynaptic GABA_B _receptors at primary afferent terminals, but not those on GABAergic and glycinergic interneurons, in the spinal cord [45]. Interestingly, a series of experiments with novel ligands at GABA_B _receptors have also suggested a functional contribution of GABA_B(1) _expressed in peripheral nociceptive neurons; e.g. α conotoxins and Rg1A peptides derived from the venom of marine Conus snails, which are currently in development for the treatment of neuropathic pain, have been shown to inhibit native calcium channel currents by the virtue of activation of GABA_B _receptors in first order neurons [47]. Thus, considerable support implicates a role for GABA_B _receptors expressed in peripheral nociceptive neurons in the endogenous modulation of nociception and pathological pain.

In this study, we deleted the primary ligand-binding subunit of metabotropic GABA receptors, GABA_B(1)_, specifically in peripheral nociceptive neurons leaving their expression in the spinal cord and brain intact. Numerous studies in cell lines as well as native tissues have demonstrated that a loss of GABA_B(1) _leads to a complete lack of ligand binding and a total loss of function of native GABA_B _receptors [28,36-38]. Therefore, based upon our findings, we infer that a conditional loss of GABA_B _receptor function in peripheral nociceptive neurons *in vivo *does not lead to significant changes in nociception and the development of pathological pain.

It is interesting to note that we have found a phenotype in firing properties of Aδ peripheral afferents, but not in C-afferents in SNS-GABA_B(1)_^-/- ^mice compared to GABA_B(1)_^fl/fl^. This might result from higher expression of GABA_B(1) _in Aδ- as compared to C-fibers. As noted previously GABA_B _receptor mRNA has been shown to be expressed in all DRG neurons [7]. However, studies on differential expression of the protein in different types of DRG neuron are lacking due to antibody specificity issues. Other possible explanation of the phenotype would be a more important role for GABA_B(1) _in Aδ-fibers in comparison to C-fibers. This hypothesis is supported by work of Sengupta et al., who observed a more prominent blockade of Aδ-fiber, than C-afferent fiber, activity upon application of systemic baclofen in pelvic nerve afferent fibers responding to isobaric colorectal distension [48].

It cannot be ruled out that compensatory changes, such as an increase in inhibition via other inhibitory transmitters and receptors, come into place to reinstate inhibition in pathological states. However, this is unlikely given that loss of GABA_B(1) _beginning at very early developmental stages, such as in classical knockout mice, does not lead to compensation of GABA_B_-mediated inhibition with respect to pain; classical GABA_B(1) _knockout mice demonstrate a prominent hyperalgesic phenotype [39]. Analyses in classical GABA_B(1) _knockout mice have confirmed that a loss of the GABA_B(1) _subunit is paralleled by a loss of all biochemical and electrophysiological GABA_B(1) _responses [25,39,49], demonstrating that GABA_B(1) _is an essential component of pre- and postsynaptic GABA_B _receptors. Directly comparing the phenotypes of global GABA_B(1) _receptor knockout mice and nociceptor-specific GABA_B(1) _knockout mice therefore leads to the inference that although GABA_B _receptors in the nervous system are important in the control of pain, these functions are likely mediated by receptors expressed in the central nervous system rather than those expressed in peripheral nociceptive neurons. However, it deserves to be noted that classical GABA_B(1) _null mutants also exhibit morphological and molecular changes in the constitution of peripheral myelin and demonstrate gate abnormalities, as revealed by very recent studies [50], thereby raising the possibility that these alterations in the periphery may have contributed to the sensory phenotype in GABA_B(1)_-deficient mice. These abnormalities would not be expected in nociceptor-specific null mutants studied here.

Interestingly, we have found a slight phenotype in the second phase of formalin response in SNS-GABA_B(1)_^-/- ^compared to GABA_B(1)_^fl/fl ^mice. There is evidence that the second phase of the formalin response depends not only on central, spinal mechanisms but also on the neural activity generated during the first phase and continuing firing activity during the second phase [51]. Therefore, the phenotype in phase IIb of the formalin response could be caused by exaggerated activation of primary afferents due to the lack of GABA_B _mediated inhibition in the first phase of the formalin test.

Experimental studies with the classical GABA_B _receptor agonist, baclofen, have implicated a therapeutic role for GABA_B _receptors in the inhibition of nociceptive hypersensitivity. However, baclofen has only found limited clinical utility in the treatment of pain. We found that systemically administered baclofen can reduce nociceptive hypersensitivity, e.g. evoked by formalin, consistent with previous reports [42,43,52]. However, analysis of nociceptor-specific GABA_B(1) _receptor mutants revealed that this anti-nociceptive activity of baclofen occurs independently of GABA_B(1) _expression in peripheral nociceptive neurons. Indeed, there is considerable evidence supporting a spinal action of baclofen in inhibiting pain; in particular, administration of baclofen attenuates mechanical allodynia in a rat spinal cord injury model, whereas a GABA_B _receptor antagonist, phaclofen, shows opposite effects [53]. Furthermore, GABA_B _receptors expressed in dorsal horn neurons have been shown to participate in the modulation of secondary hyperalgesia in monoarthritic rats, which is reduced by intrathecal injection of baclofen [16]. However, some studies have also suggested a presynaptic locus of action of baclofen. For example, electrophysiological studies have suggested inhibition of neurotransmitter release from presynaptic terminals via baclofen-mediated activation of GABA_B _receptors [43,45]. However, the consequences of baclofen-induced inhibition of presynaptic neurotransmitter release from nociceptive afferents in the spinal cord are somewhat tampered by the consequential reduction of GABAergic and glycinergic synaptic transmission onto substantia gelatinosa neurons, which are typically also activated by the glutamatergic inputs coming in via peripheral afferents [54].

Indeed, GABA_B _receptors are also widely distributed in a variety of brain regions which play an important role in the modulation of pain, e.g. the rostral agranular insular cortex, a cortical area which is constantly activated by painful stimuli [55]. Furthermore, it has been shown that a local increase of GABA_B _concentrations in higher brain centres results in lasting bilateral analgesia [56]. Thus, the locus of baclofen action remains unclear.

Our analyses suggest that baclofen-induced inhibition of anti-nociception, particularly at doses which do not cause motor impairment, is not mediated by GABA_B _receptors on presynaptic nerve terminals. A detailed analysis of baclofen-induced anti-nociception is considerably hindered by the marked motor impairment caused by baclofen at higher doses. We observed that intraplantar injection of baclofen in the hind paw did not lead to anti-nociception at low doses (data not shown); doses which evoked anti-nociception upon intraplantar administration were accompanied by a marked impairment of motor function and paralysis. Based upon these pieces of evidence, we conclude that peripheral nociceptive neurons are not the primary locus of baclofen action in the modulation of pain.

## Conclusion

In summary, this study clarifies a long-standing question in the field of GABAergic modulation of nociception, namely the contribution of presynaptic GABA_B _receptors in primary afferent nociceptive neurons. The use of genetic tools to specifically delete GABA_B _receptors in DRG neurons, while leaving their expression in the spinal cord and brain intact, revealed that GABA_B _receptors in primary nociceptive neurons do not play a major role in the modulation of pain. Furthermore, our results suggest that anti-nociceptive effects evoked by GABA_B _receptor agonists are not mediated by receptors in peripheral nociceptive neurons but by receptors in the central nervous system. Thus, our results suggest that it would be advantageous to focus on the central nervous system when harnessing the GABA_B _receptor system for pain management.

## List of abbreviations used

CFA: complete Freud's adjuvant; DRG: dorsal root ganglia; GABA: γ-aminobutyric acid; PBS: phosphate buffered saline; PWL: paw withdrawal latency; SNI: spared nerve injury.

## Competing interests

The authors declare that they have no competing interests.

## Authors' contributions

VG performed a large portion of the experiments and analyzed data; NA, IT and SB performed experiments; BB provided the GABA_B(1)_^fl511/fl511 ^mice; RK designed and supervised the study and helped with the writing of the manuscript; MK performed a large portion of experiments, analyzed data and wrote the manuscript. All authors read and approved the final manuscript.
